# A bibliometric analysis of interstitial cells of Cajal research

**DOI:** 10.3389/fmed.2024.1391545

**Published:** 2024-05-20

**Authors:** Pengyu Li, Yadan Xiao, Lan Zhou, Xuyuan Zhang, Yin Xu, Xiaojuan Wang, Menglong Zou, Xuan Guo

**Affiliations:** ^1^School of Integrated Chinese and Western Medicine, Hunan University of Chinese Medicine, Changsha, China; ^2^Integrated Traditional Chinese and Western Medicine Department, The Third Hospital of Changsha, Changsha, China; ^3^Department of Gastroenterology, The First Hospital of Hunan University of Chinese Medicine, Changsha, China; ^4^Science & Technology Innovation Center (National Key Laboratory Cultivation Base of Chinese Medicinal Powder & Innovative Medicinal Jointly Established by Province and Ministry), Hunan University of Chinese Medicine, Changsha, China

**Keywords:** interstitial cells of Cajal, CiteSpace, VOSviewer, bibliometrics, visualization

## Abstract

**Objective:**

The significance of interstitial cells of Cajal (ICC) in the gastrointestinal tract has garnered increasing attention. In recent years, approximately 80 articles on ICC have been published annually in various journals. However, no bibliometric study has specifically focused on the literature related to ICC. Therefore, we conducted a comprehensive bibliometric analysis of ICC to reveal dynamic scientific developments, assisting researchers in exploring hotspots and emerging trends while gaining a global perspective.

**Methods:**

We conducted a literature search in the Web of Science Core Collection (WoSCC) from January 1, 2013, to December 31, 2023, to identify relevant literature on ICC. We employed bibliometric software, namely VOSviewer and CiteSpace, to analyze various aspects including annual publication output, collaborations, research hotspots, current status, and development trends in this domain.

**Results:**

A total of 891 English papers were published in 359 journals by 928 institutions from 57 countries/regions. According to the keyword analysis of the literature, researchers mainly focused on “c-Kit,” “expression,” “smooth muscle,” and “nitric oxide” related to ICC over the past 11 years. However, with “SIP syncytium,” “ANO1,” “enteric neurons,” “gastrointestinal stromal tumors (GIST),” and “functional dyspepsia (FD),” there has been a growing interest in the relationship between ANO1, SIP syncytium, and ICC, as well as the role of ICC in the treatment of GIST and FD.

**Conclusion:**

Bibliometric analysis has revealed the current status of ICC research. The association between ANO1, SIP syncytium, enteric neurons and ICC, as well as the role of ICC in the treatment of GIST versus FD has become the focus of current research. However, further research and collaboration on a global scale are still needed. Our analysis is particularly valuable to researchers in gastroenterology, oncology, and cell biology, providing insights that can guide future research directions.

## 1 Introduction

In 1889, the Spanish neuroanatomist Santiago Ramon y Cajal (1852–1934) made an initial groundbreaking discovery of small individual nerve ganglion cells in the gastrointestinal tissues of mammals. These cells were morphologically characterized as spindle-shaped stellate cells and named “interstitial cells of Cajal (ICC)” (1). However, at that time, only morphological techniques were available to identify ICC, and their physiological functions remained elusive for many years. It was not until the 1990s that the tyrosine kinase receptor Kit (c-Kit), also known as CD117 or the stem cell factor receptor, was identified as the primary marker for ICC in pathological specimens (2, 3). This discovery marked a significant breakthrough in the field. Over time, ICC has emerged as a focal point in the fields of physiology and medicine (4, 5), with their role in the gastrointestinal tract and other organs attracting considerable attention due to their association with various diseases and physiological processes (6, 7). ICC possesses distinctive ultrastructural features and electrophysiological properties, playing a crucial role in regulating smooth muscle contraction and coordinating gastrointestinal motility. They are commonly referred to as the “pacemaker cells” of the gastrointestinal tract (8). Research has shown that ICC display distinct functionalities across different subtypes (9). Intramuscular interstitial cells (ICC-IM) modulate neurotransmitter responses (10), and myenteric interstitial cells (ICC-MY) serve as pacemakers by generating slow waves that influence smooth muscle contractions (11). On the other hand, submucosal interstitial cells (ICC-SM) coordinate the regulation of secretions and reflexes within the mucosal and submucosal layers (12), while septal interstitial cells (ICC-SEP) function as a supportive network, maintaining the structural integrity of the gastrointestinal wall (13, 14). However, as time passes, the complexity and diversity of ICC research have steadily increased, extending to multiple systems, such as the nervous, digestive, and urinary systems, among others (9, 15, 16). Consequently, there is a need for a comprehensive methodology to fully understand their developmental trajectories and impact.

Bibliometrics is a subfield of informatics that involves the use of mathematical and statistical methods for quantitative and qualitative analysis of published scholarly literature (17–19). Integrating temporal and spatial dimensions into bibliometric analysis can provide new insights into knowledge development and academic records (20). This approach focuses on countries, institutions, journals, authors, and keywords associated with a specific field of study, measuring the profile of the field, partnerships, and overall scholarly output. It aims to provide readers with objective view of trends and frontiers in the field with the aim of assessing the characteristics and trends of a specific research area (21, 22). Furthermore, it helps to analyze the co-occurrence and impact of a given field, making it an indispensable tool for assessing the quality and impact of scholarly work (23). Despite the growing utilization of bibliometrics in various fields, there remains a notable gap in bibliometric research specifically focused on ICC. Addressing this gap, the present study capitalizes on the Web of Science^™^ Core Collection (WoSCC) to collect pertinent bibliometric data concerning related to ICC research from 2013 to 2023. Using CiteSpace and VOSviewer tools (24, 25), knowledge maps are generated to help illustrate scientific knowledge and various relationships, providing valuable insights for future research endeavors.

## 2 Materials and methods

### 2.1 Data sources and search strategy

**Figure 1:**
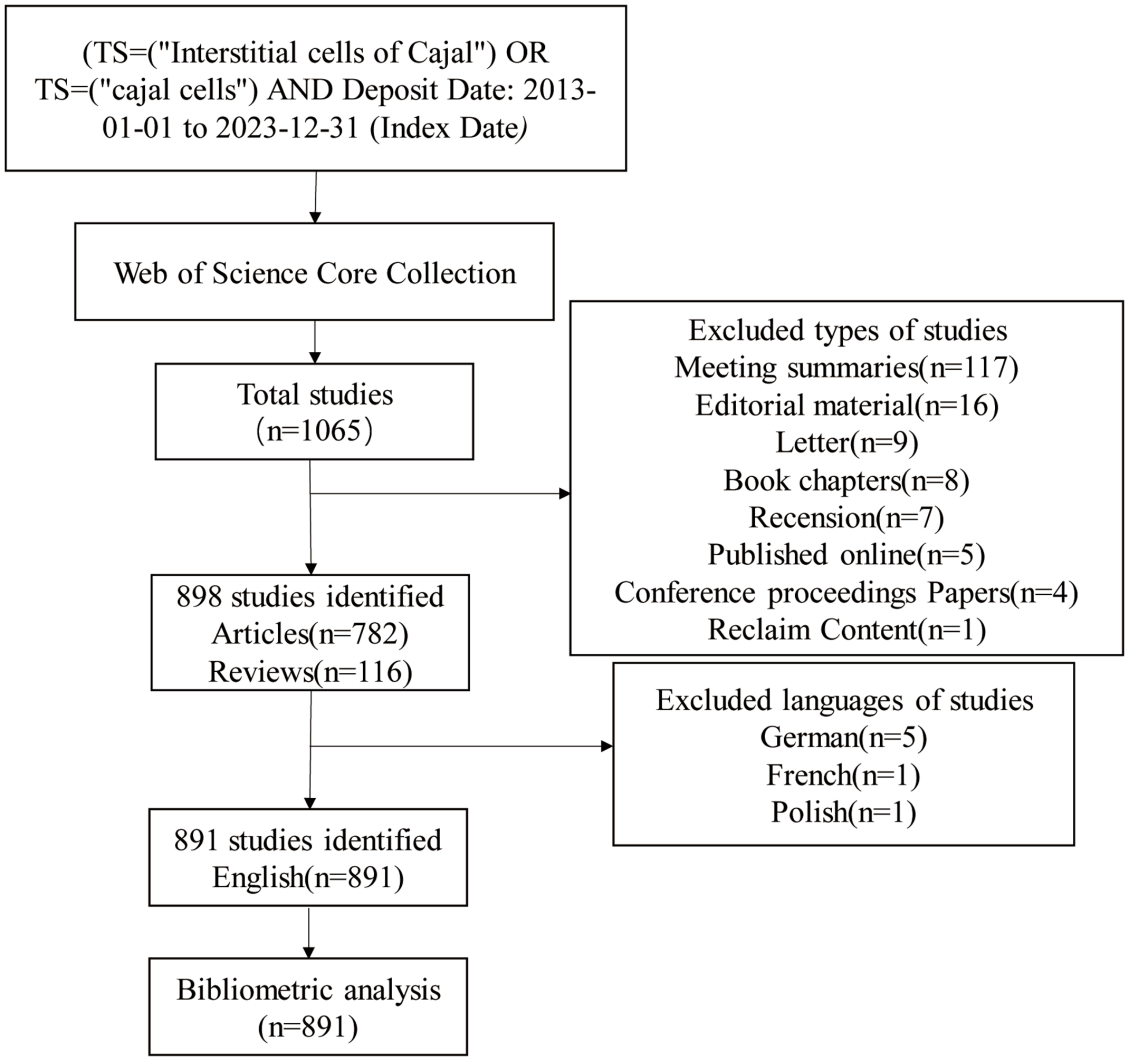
The flowchart of literature selection.

In this study, we utilized the comprehensively recognized and standardized WoSCC as our primary database for retrieving literature on ICC (26). Compared to Scopus, the accuracy of document type labels in WoSCC is higher (27). Our search strategy was (((TS = (“Interstitial cells of Cajal”)) OR TS = (“Cajal cells”))), with a specified time frame from January 1, 2013, to December 31, 2023. No ethical approval was required as our research did not involve animal experiments or human trials. We selected research articles and reviews that met our inclusion criteria and were written in English to ensure professionalism and accuracy. Non-English articles, other document types, and publications outside the specified time range were excluded. The collected data included complete bibliographic records and citation information, all stored in plain text format. To maintain accuracy and timeliness, all retrieval and collection work was finalized on January 12, 2024, thus minimizing the impact of subsequent database updates. The primary objective of this study was to conduct a systematic literature search on a specific topic and establish stringent linguistic standards for subsequent analytical processes. A visual representation of the retrieval process is shown in Figure 1.

### 2.2 Data analysis

The CiteSpace software developed by Professor Chao-Mei Chen at American University can be used to visualize and analyze trends and patterns in the scientific literature, which can help to predict research trends in a particular discipline or field over a specific period of time (28, 29). The parameters used for analysis in CiteSpace (version 6.1.R6) were as follows: a link retaining factor of 3.0; a time span from 2013 to 2023 with one slice per year; node types including Reference and Keyword; links with strength measured by cosine and scope within slices; and a selection criteria with a g-index scale factor of 25. In addition to CiteSpace, we used VOSviewer (version 1.6.16) to visualize and analyze the co-occurrence of countries, institutions, author distributions, and keywords. For co-occurrence analysis of keywords, adjustments were made using the Pajek program to enhance clarity of relationships. Geographical visualization of country and institution distribution was performed using Scimago Graphica. In addition, the History of Cite software (HisCite, v2.1) developed by Garfield was utilized for drawing citation maps to examine links between different scholarly works and to identify important publications. Burst detection of keywords and references was generated using CiteSpace.

Furthermore, we employed Microsoft Office Excel 2021 to conduct statistical analyses on the distribution of journals by publication year, authors, countries, institutions, citation rankings, and journal impact factors. We assessed the scientific impact of journals based on the 2023 Journal Citation Reports (JCR). This assessment specifically involved using the journal category Impact Factors (IF) and quartile rankings to gauge their significance in the field.

## 3 Results

### 3.1 Analysis of publications

**Figure 2:**
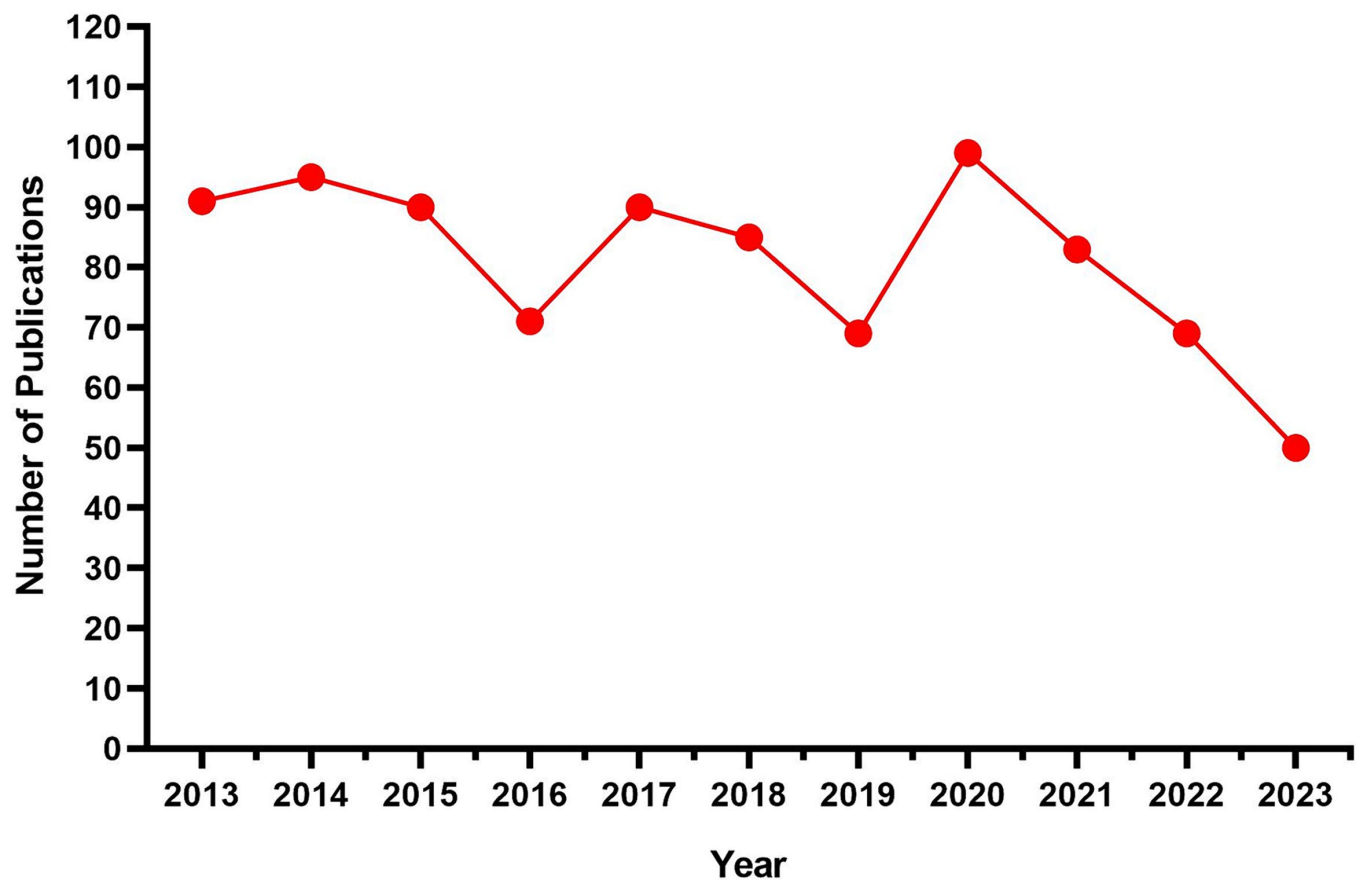
Annual trends in publications.

The annual publication output often serves as an indicator of the development status within a field (28). Figure 2 depicts the publication trends in this particular field from 2013 to 2023. Over this period, there has been a noticeable fluctuation in the number of publications. Nonetheless, overall, this field has consistently maintained a substantial level of scholarly output. These findings suggested that researchers demonstrated a lasting commitment to the investigation of ICC throughout the past 11 years. These findings not only highlight the level of activity in the field, but also demonstrate the interest and motivation of researchers to delve deeper into the complex issues involved in ICC investigations.

### 3.2 Analysis of countries/regions

A total of 891 publications on ICC were authored by 928 institutions from 57 countries/regions. The top 10 countries/regions and institutions in terms of productivity are detailed in Tables 1, 2. Our findings illustrated that China (231, 25.93%) exhibited the highest productivity, followed by the United States (224, 25.1%), South Korea (87, 9.76%), Germany (37, 4.15%), and Italy (36, 4.04%). The most active affiliated institutions included the University of Nevada (70, 7.86%), Mayo Clinic (52, 5.84%), Pusan National University (47, 5.27%), the University of Auckland (43, 4.83%), and McMaster University (30, 3.37%). Despite the numerical dominance of China and the United States, our analysis of the overall network strength among countries/regions and organizations shows that the United States and Mayo Clinic have significant influence in this area. This reflects the depth and leadership of these countries and organizations in research, cooperation, and influence in the field of ICC investigations.

**Table 1:** The 10 countries/regions with the highest number of outputs and the highest degree of cooperation.

Rank	Country	Counts (%)	Co-authorship country/region	Total link strength
1	China	231 (25.93%)	USA	146
2	USA	224 (25.14%)	China	48
3	South Korea	87 (9.76%)	New Zealand	43
4	Germany	37 (4.15%)	Germany	33
5	Italy	36 (4.04%)	South Korea	29
6	Japan	36 (4.04%)	Italy	25
7	New Zealand	33 (3.70%)	United Kingdom	25
8	Canada	27 (3.03%)	Japan	21
9	United Kingdom	26 (2.92%)	Belgium	21
10	Belgium	25 (2.81%)	Canada	15

**Table 2:** The 10 institutions with the highest number of outputs and the highest degree of cooperation.

Rank	Institution	Counts (%)	Co-authorship institution	Total link strength
1	Univ Nevada	70 (7.86%)	Mayo Clin	113
2	Mayo Clin	52 (5.84%)	Univ Auckland	78
3	Pusan Natl Univ	47 (5.27%)	Pusan Natl Univ	71
4	Univ Auckland	43 (4.83%)	Univ Nevada	70
5	McMaster Univ	30 (3.37%)	Vanderbilt Univ	69
6	Vanderbilt Univ	28 (3.14%)	Seoul Natl Univ	60
7	Seoul Natl Univ	27 (3.03%)	Univ Louisville	50
8	Huazhong Univ Sci & Technol	20 (2.24%)	Stanford Univ	41
9	Wuhan Univ	19 (2.13%)	Johns Hopkins Univ	38
10	Shanghai Jiao Tong Univ	18 (2.02%)	Carol Davila Univ Med & Pharm	37

**Figure 3:**
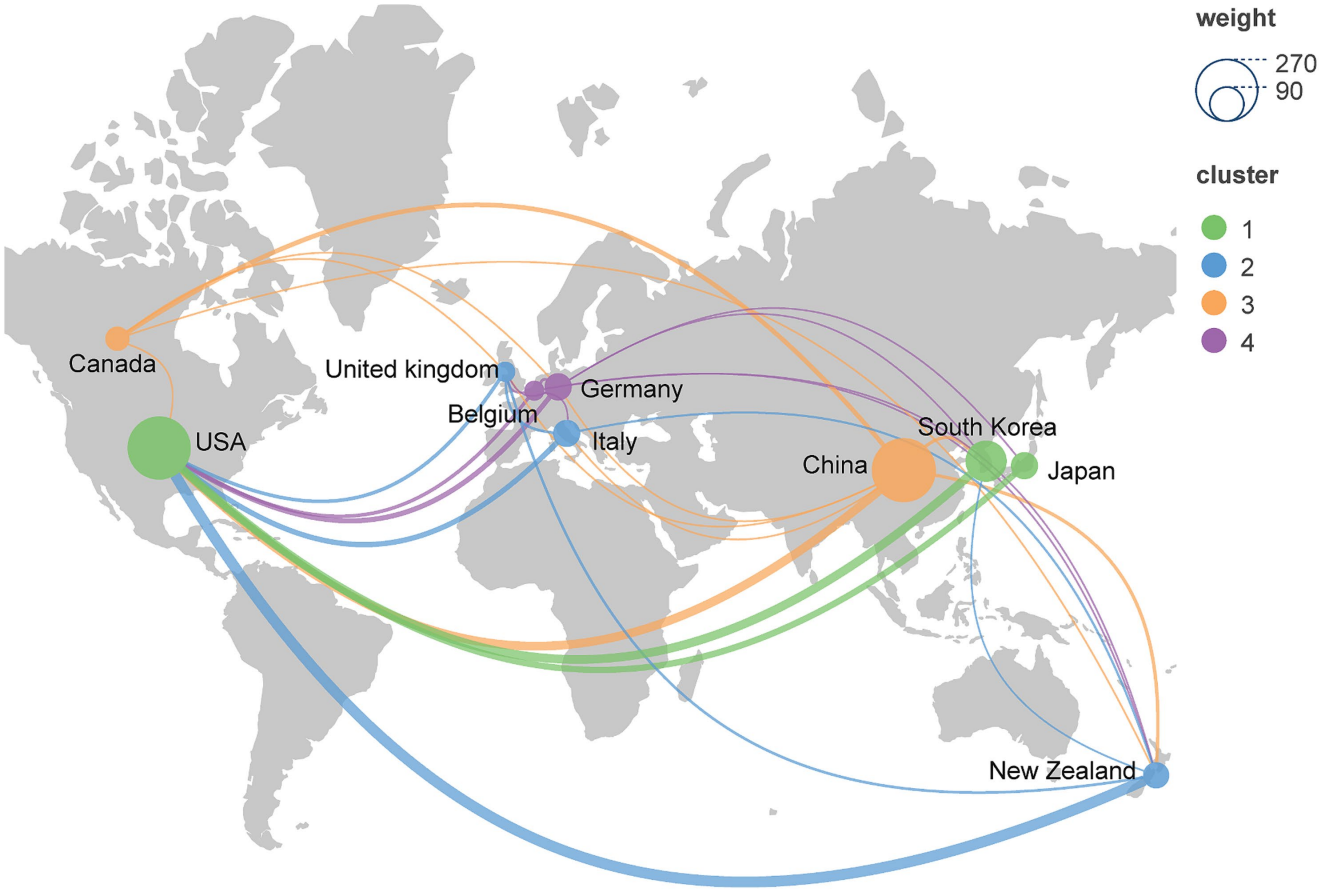
Geographic bibliometric map based on a network of co-authorship relationships in the top 10 countries in terms of number of articles published.

Figure 3 illustrates a bibliometric map of geography, showcasing the collaborative authorship network among the top 10 countries with the highest number of published articles. Notably, China and the United States made the most significant contributions, followed by other noteworthy contributors such as South Korea, Germany, and Italy. In addition to the number of collaborations, the quality of collaboration between these countries is also noteworthy. In addition, articles in this field show a commendable level of citation quality, which reflects their far-reaching impact and scholarly value.

**Figure 4:**
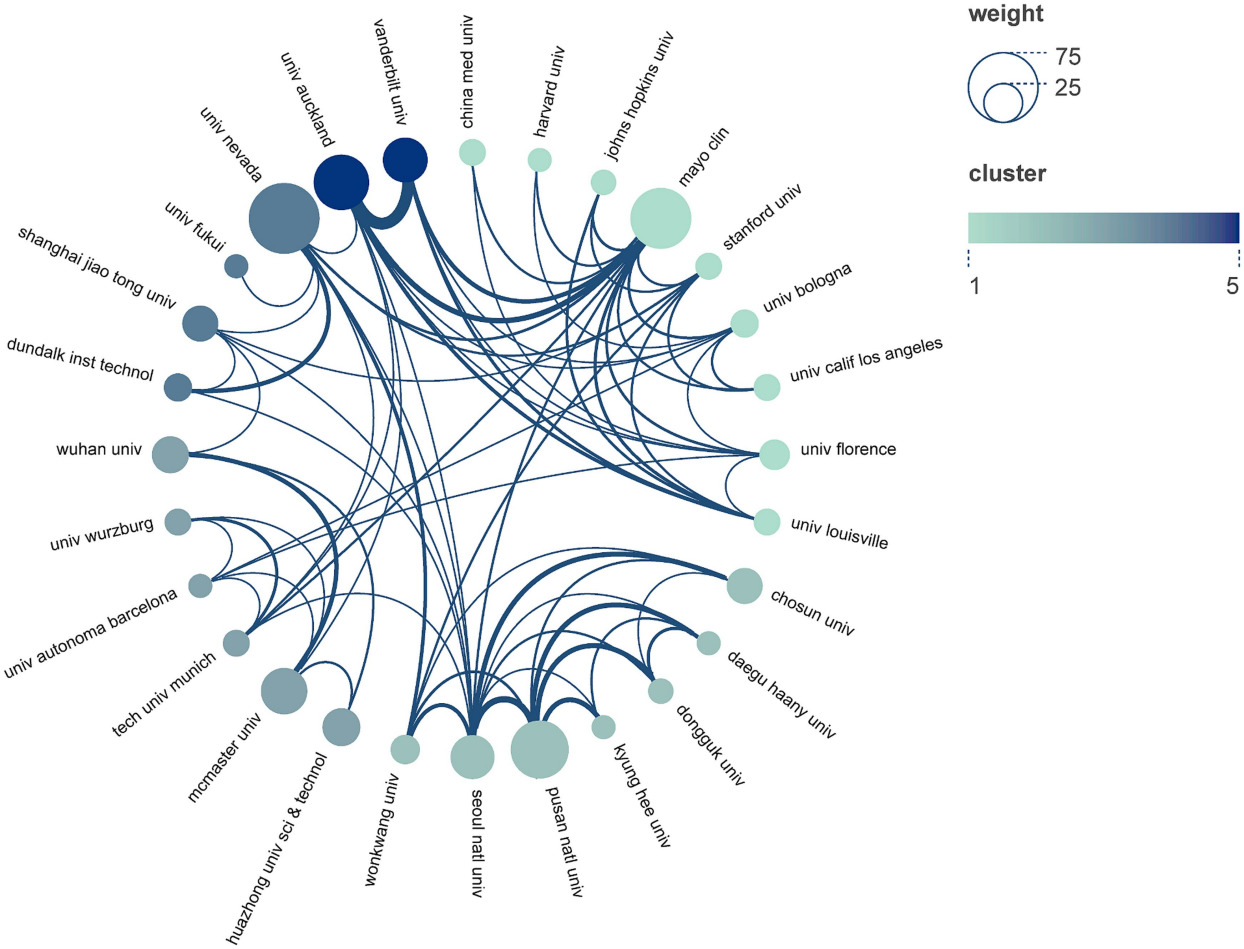
Institutions collaboration map.

In Figure 4, a collaborative map portrays the relationships between countries/regions and institutions. The size of each node reflects the number of documents, while the thickness and color of the connecting lines indicate the extent of collaboration between them. It is evident that the University of Nevada and Mayo Clinic engage in close collaboration with numerous institutions. Furthermore, the University of Auckland and Vanderbilt University exhibited a robust partnership in terms of institutional collaborations. Several research institutions, including Pusan National University, McMaster University, and Seoul National University, actively participated in collaborative endeavors.

### 3.3 Distribution of journals

A total of 359 journals published the articles related to this research. An overview of the top 10 most relevant journals in terms of these publications is summarized in Table 3. The majority of these journals boast an IF of 3 or higher, and they predominantly fall into the Q3 category or higher according to the Journal Citation Reports (JCR) categorization. Notably, Neurogastroenterology and Motility, American Journal of Physiology-Gastrointestinal and Liver Physiology, and PLoS One are the top three journals that have published the greatest number of articles related to this field of study. This reflects the leading position of these journals in the field and their important role in promoting scholarly exchange and knowledge dissemination.

**Table 3:** The top 10 journals and co-cited journals.

Rank	Journal	Count	Total citations	IF (2023)	JCR	Co-cited journal	Total citations	IF (2023)	JCR
1	Neurogastroenterology and Motility	63	1,238	6.6	Q3	Journal of Physiology-London	2,805	5.5	Q2
2	American Journal of Physiology-Gastrointestinal and Liver Physiology	32	567	4.5	Q3	Gastroenterology	2,399	29.4	Q1
3	PLoS One	26	488	3.7	Q3	Neurogastroenterology and Motility	2,378	3.5	Q3
4	Journal of Neurogastroenterology and Motility	25	333	3.4	Q3	American Journal of Physiology-Gastrointestinal and Liver Physiology	1,913	4.5	Q3
5	Journal of Physiology-London	23	521	5.5	Q2	Journal of Cellular and Molecular Medicine	744	5.3	Q2
6	World Journal of Gastroenterology	22	388	4.3	Q3	Proceedings of the National Academy of Sciences of the United States of America	592	11.1	Q1
7	Journal Of Cellular and Molecular Medicine	18	534	5.3	Q2	Gut	591	24.5	Q1
8	Gastroenterology	13	604	29.4	Q1	Cell and Tissue Research	589	3.6	Q3
9	Evidence-Based Complementary and Alternative Medicine	13	116	—	—	Nature	575	64.8	Q1
10	International Journal of Molecular Sciences	10	143	5.6	Q2	American Journal of Physiology-Cell Physiology	524	5.5	Q2

Journal co-citation is a method employed to examine citation relationships and influences among different academic journals. Its principal objective is to aid researchers and academic institutions in comprehending the interconnections between journals and assessing their impact and quality. Upon analyzing Table 3, it was clear that three academic journals received over 2000 citations each: Journal of Physiology-London, Gastroenterology, and Neurogastroenterology and Motility. Furthermore, among these journals, seven have an IF greater than 5, further highlighting their high level of academic quality and research influence.

**Figure 5:**
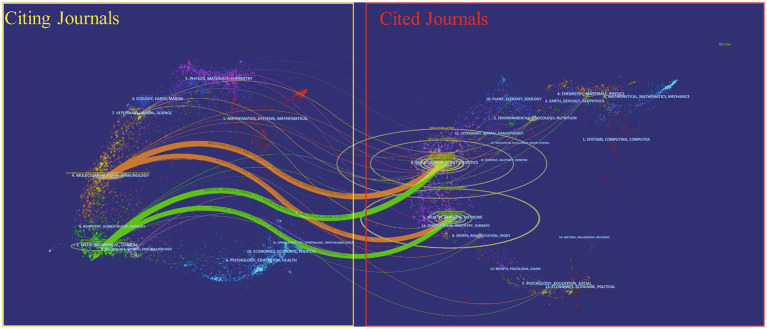
The dual-map overlay of journals.

A dual-mapping overlay of journals is shown in Figure 5, demonstrating the thematic distribution of academic journals, changes in citation trajectories, and shifts in research focus. The graph accurately depicts the distribution of individual academic journals and vividly shows the relationships between journals (where the colored paths represent citation relationships) (30). The left side of the figure represents the citing journals, while the right side represents the cited journals. Labels on the figure indicate the various disciplines covered by the journals, and the colored pathways highlight the citation relationships. Notably, four prominent pathways can be observed. Two orange citation pathways indicated that Molecular Biology and Genetics journals, as well as Health, Nursing, and Medicine journals, were frequently cited by Molecular/Biology/Immunology journals. Additionally, two green citation pathways demonstrated that Molecular Biology and Genetics journals, along with Health, Nursing, and Medicine journals, were frequently cited by Medicine/Medical/Clinical journals. These findings not only contribute to understand the relationships among different academic journals, but also help to reveal intersections and trends between various subject areas, thus providing valuable insights into academic research and interdisciplinary collaboration.

### 3.4 Distribution of authors

In this specific research domain, 4,115 researchers made notable contributions. Table 4 provides a comprehensive snapshot of the top 10 researchers who have made outstanding contributions and garnered significant citations. Notably, among these esteemed scholars, four were affiliated with the University of Nevada, while two were affiliated with the Mayo Clinic. Sanders, Kenton M., Kim, Byung Joo, and Farrugia, Gianrico emerge as three of the most prolific contributors in this field.

**Table 4:** The top 10 authors and co-cited authors.

Rank	Author	Count (%)	Co-cited author	Citation
1	Sanders, Kenton M.	52 (5.84%)	Sanders, Kenton M.	1,480
2	Kim, Byung Joo	41 (4.60%)	Ward, Sean M.	960
3	Farrugia, Gianrico	29 (3.25%)	Farrugia, Gianrico	888
4	Huizinga, Jan D.	27 (3.03%)	Koh, Sang Don	671
5	Cheng, Leo K.	26 (2.92%)	Du, Peng	566
6	Ward, Sean M.	25 (2.81%)	Cheng, Leo K.	478
7	Drumm, Bernard T.	23 (2.58%)	Huizinga, Jan D.	461
8	Du, Peng	22 (2.47%)	Gibbons, Simon J.	451
9	Baker, Salah A.	22 (2.47%)	Saur, Dieter	405
10	Gibbons, Simon, J.	18 (2.02%)	O’grady, Gregory	391

Co-citation analysis of authors refers to the situation where two authors’ papers are cited by a third author simultaneously (17). The evaluation of researchers’ co-citation patterns highlighted individuals who exerted a substantial impact on the field. Table 4 presents the top 10 most co-cited scholars. In terms of citation counts, Sanders, Kenton M. held the leading position with 1,480 citations, further reinforcing his prominence as the most prolific author in terms of publications. Following closely behind were Ward, Sean M. (960 citations), Farrugia, Gianrico (888 citations), Koh, Sang Don (671 citations), and Du, Peng (566 citations).

**Figure 6:**
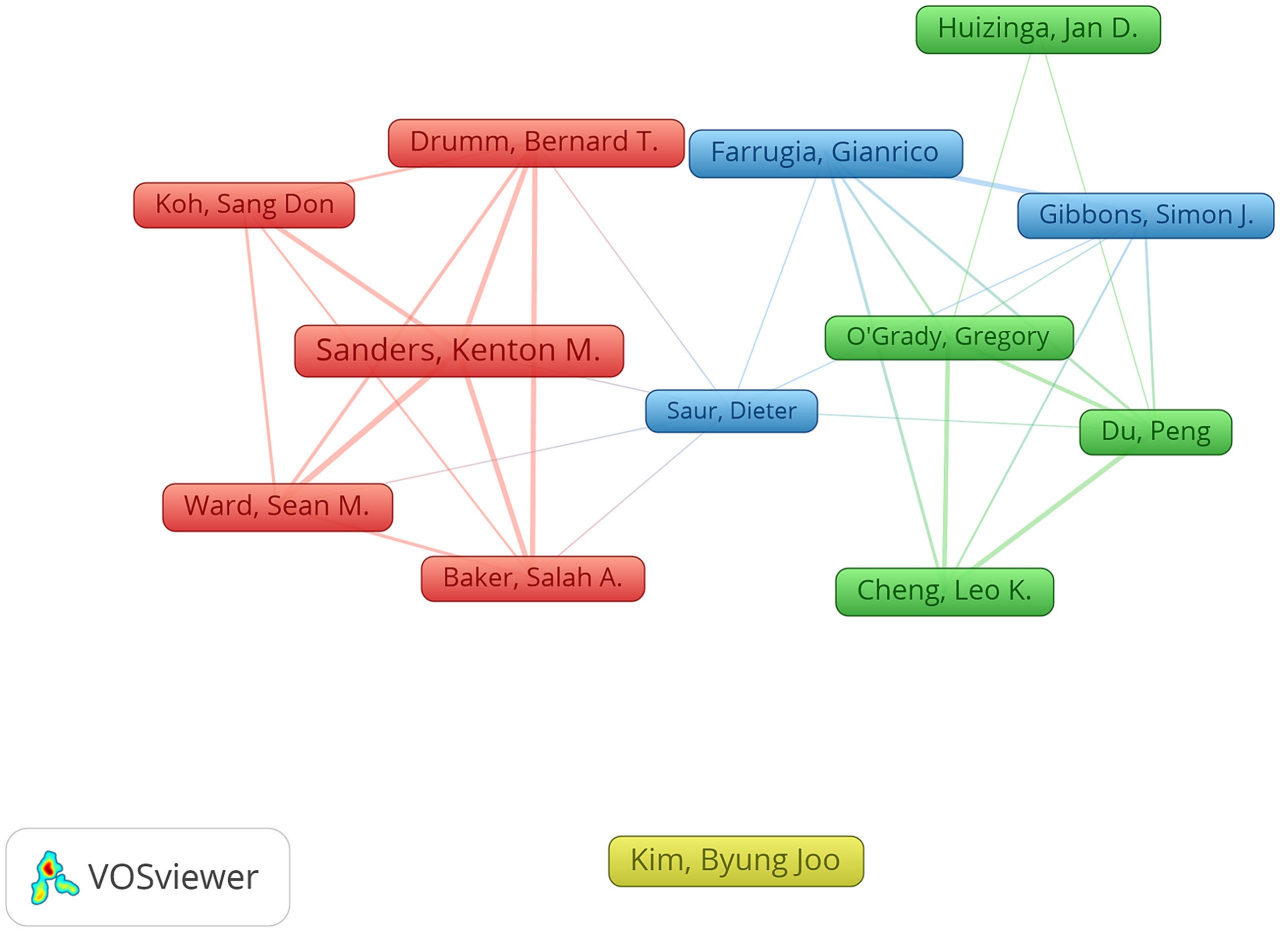
Co-authorship network map.

Notably, this research domain attracted considerable attention from multiple academic teams. Figure 6 illustrates the top 10 authors with the highest number of publications and citations. Each node within the figure represents an individual researcher, with the size of the nodes indicating the number of publications by each researcher. The connections between nodes represent collaborative relationships among these researchers. Note that different clusters in the graph represent different collaborative groups. Our analysis reveals active participation in ICC research among the most contributing teams. However, the collaborative relationships between these teams are relatively limited, and there is significant room to optimize and make the best use of the available resources.

### 3.5 Keyword co-occurrence analysis

**Figure 7:**
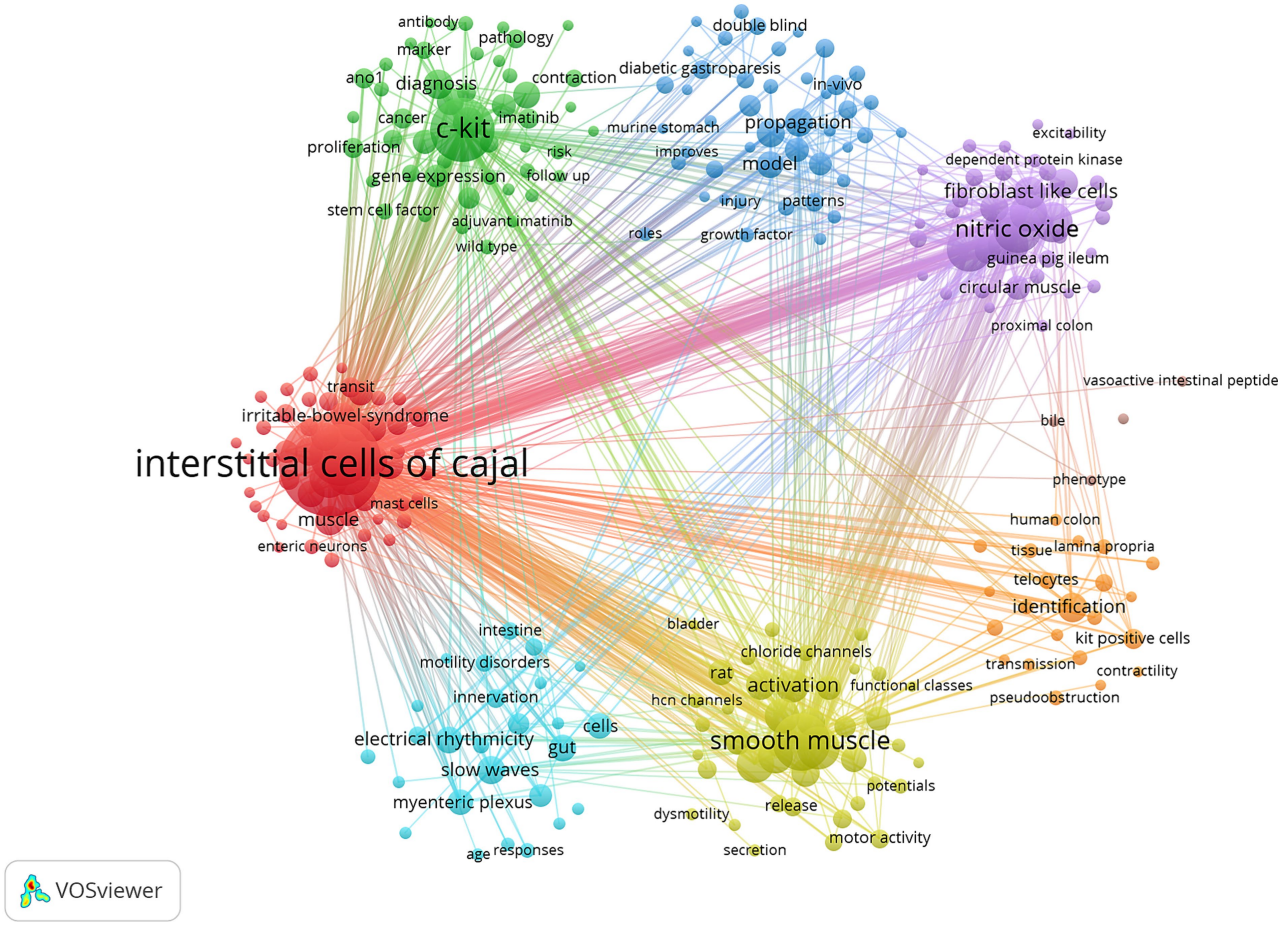
Keywords co-occurrence visualization map.

The keywords used in an article serve as indicators of the research theme and can effectively identify research hotspots and frontiers within a specific field. To achieve this, we utilized VOSviewer software to create a co-occurrence network visualization (Figure 7), which comprises 277 high-frequency keywords (occurring more than 5 times). This visualization offers a graphical representation of their relationships based on their co-occurrence frequencies in the literature. Furthermore, Table 5 presents a compilation of the top 30 keywords that are closely associated with ICC. In addition to the prominent term “Interstitial cells of Cajal,” keywords such as “c-Kit,” “expression,” “smooth muscle,” and others prominently appear in the abstracts and titles of articles.

**Table 5:** The top 30 keywords related to ICC.

Rank	Keyword	Count	Total link strength	Rank	Keyword	Count	Total link strength
1	interstitial cells of Cajal	384	1,763	16	activation	40	227
2	c-Kit	150	749	17	fibroblast-like cells	38	244
3	expression	135	704	18	mutation	36	174
4	smooth muscle	121	680	19	neurotransmission	34	198
5	nitric-oxide	84	483	20	gastrointestinal motility	34	174
6	motility	75	377	21	diagnosis	33	139
7	gastrointestinal tract	74	420	22	muscle	33	139
8	pacemaker activity	67	411	23	identification	32	175
9	enteric nervous system	60	336	24	guinea pig	31	173
10	smooth muscle cell	60	329	25	currents	31	168
11	mouse	55	285	26	inhibitory neurotransmission	30	201
12	small intestine	53	314	27	slow waves	30	175
13	receptor	52	255	28	propagation	30	173
14	murine small intestine	43	245	29	channels	30	165
15	mechanism	43	234	30	Ca^2+^	29	161

The co-occurrence network classified keywords into distinct clusters, each distinguished by different colors. We identified 7 clusters. The red category primarily encompasses the physiological functions of ICC, such as their roles in intestinal transport and muscle activity. The green category mainly consists of molecular markers related to ICC. The blue category focuses on clinical studies related to ICC, with keywords such as “*in-vivo*” and “double blind,” suggesting a focus on evaluating the impact of therapeutic interventions on ICC function, especially in the context of diabetic gastroparesis. The light blue category primarily focuses on the electrophysiological aspects of ICC, involving keywords like “electrical rhythmicity” and “slow waves,” indicating a greater emphasis on the role of ICC in the electrical activity of the gastrointestinal tract and its importance as pacemaker cells The orange category mainly deals with the pathological identification of ICC, with keywords like “kit positive cells” and “human colon,” indicating research into ICC in different types of cells and the human colon. The yellow category primarily explores the relationship between ICC and gastrointestinal motility, involving keywords such as “smooth muscle” and “motility,” suggesting that research may focus on the role of ICC in regulating smooth muscle contraction and intestinal dynamics. The purple category mainly focuses on the biophysical properties of ICC, such as “nitric oxide” and “excitability,” suggesting that the role of ICC in intestinal excitatory and inhibitory signaling may be investigated.

Additionally, certain keywords displayed strong link strengths despite their lower frequencies, suggesting emerging research areas or topics that have not yet received extensive exploration but deserve closer attention from researchers. For instance, keywords such as “pacemaker activity” and “motility” exhibited lower occurrence frequencies but demonstrated significant link strengths, implying their relevance to ICC research, even though they may not have received substantial investigation thus far.

### 3.6 Analysis of HisCite literature

**Figure 8:**
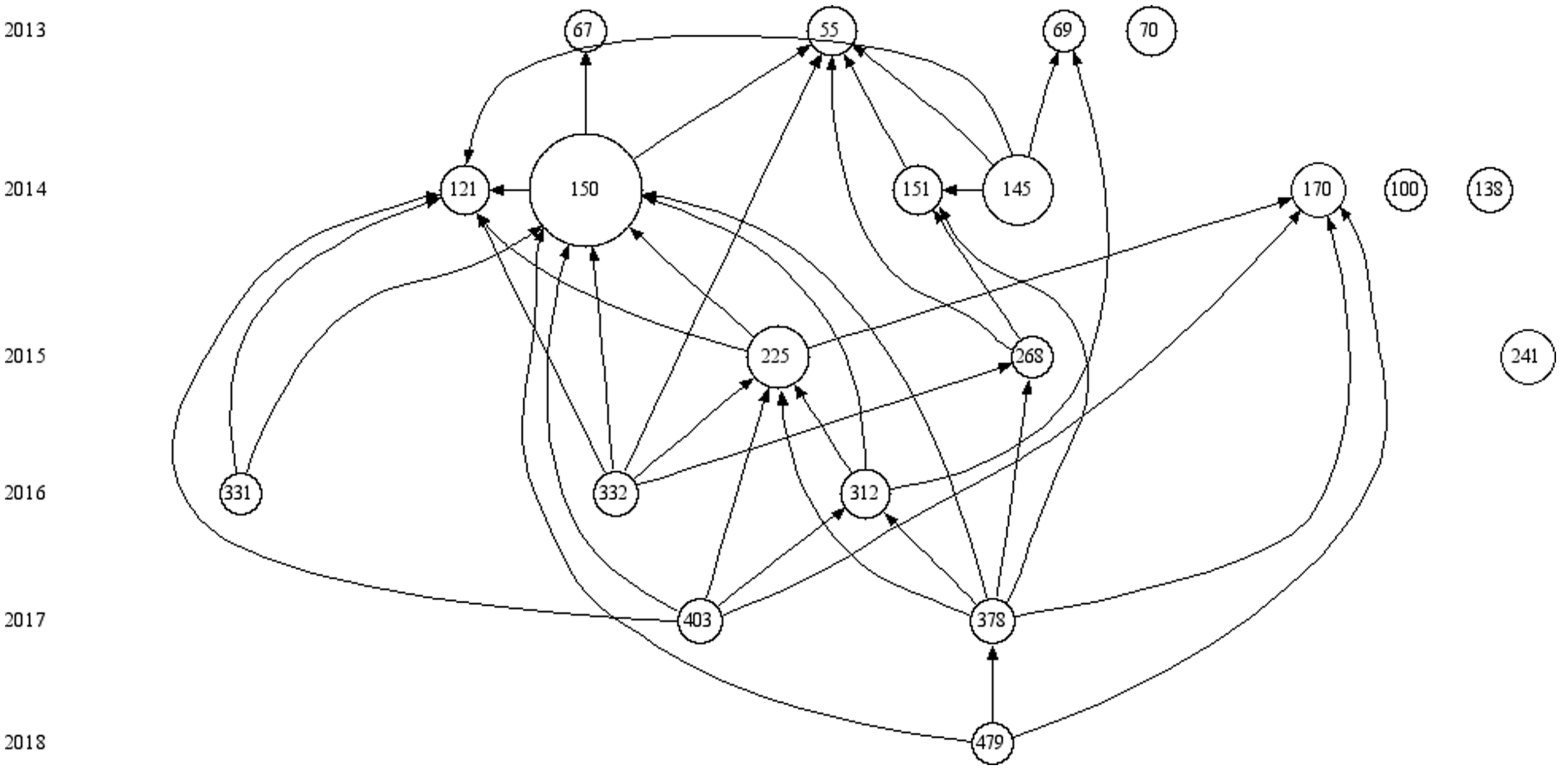
Network of the top 20 ICC research articles based on LCS ranking. Each of the 20 circles in the figure represents a key literature reference, with the central number denoting its position in the database, corresponding to the ID sequence in Table 6. Circle size indicates citation frequency—larger circles correspond to higher citation rates. Arrows between circles show citation relationships.

To analyze ICC research progression, we identified the top 20 articles based on Local Citation Score (LCS) from HisCite, as depicted in Figure 8 and Table 6. The LCS is the number of citations a paper receives in a given academic field, and the sum of its indices reflects its influence and visibility in that field (31).

**Table 6:** The detailed information on the top 20 ICC research articles based on LCS.

Rank	ID	Title	LCS
1	150	Interstitial cells: regulators of smooth muscle function	136
2	145	The significance of interstitial cells in neurogastroenterology	53
3	225	Intracellular Ca^2+^ release from endoplasmic reticulum regulates slow wave currents and pacemaker activity of interstitial cells of Cajal	44
4	170	Ano1, a Ca^2+^-activated Cl^−^ channel, coordinates contractility in mouse intestine by Ca^2+^ transient coordination between interstitial cells of Cajal	34
5	241	Loss of interstitial cells of Cajal and patterns of gastric dysrhythmiain patients with chronic unexplained nausea and vomiting	31
6	151	Mitochondrial calcium handling within the interstitial cells of Cajal	28
7	121	Expression and function of a T-type Ca^2+^ conductance in interstitial cells of Cajal of the murine small intestine	28
8	55	Cell-specific deletion of nitric oxide-sensitive guanylyl cyclase reveals a dual pathway for nitrergic neuromuscular transmission in the murine fundus	26
9	312	Spontaneous Ca^2+^ transients in interstitial cells of Cajal located within the deep muscular plexus of the murine small intestine	26
10	70	Interstitial cells of Cajal in the normal human gut and in Hirschsprung disease	26
11	332	Regulation of gastrointestinal smooth muscle function by interstitial cells	25
12	378	Conditional genetic deletion of Ano1 in interstitial cells of Cajal impairs Ca^2+^ transients and slow waves in adult mouse small intestine	25
13	403	Clustering of Ca^2+^ transients in interstitial cells of Cajal defines slow wave duration	25
14	138	The possible roles of hyperpolarization-activated cyclic nucleotide channels in regulating pacemaker activity in colonic interstitial cells of Cajal	24
15	69	Differential expression of genes related to purinergic signaling in smooth muscle cells, PDGFRα-positive cells, and interstitial cells of Cajal in the murine colon	20
16	268	Nitrergic signalling via interstitial cells of Cajal regulates motor activity in the murine colon	20
17	479	The cells and conductance mediating cholinergic neurotransmission in the murine proximal stomach	19
18	331	Nitric oxide-induced oxidative stress impairs the pacemaker function of murine interstitial cells of Cajal during inflammation	19
19	67	Telocytes express PDGFRα in the human gastrointestinal tract	18
20	100	Decreased SCF/c-Kit signaling pathway contributes to loss of interstitial cells of Cajal in gallstone disease	18

Our analysis highlighted Sanders, Kenton M.’s work as having high citation rates, with a focus on reviewing interstitial cells in smooth muscle, particularly ICC and PDGFRα(+) cells in the gastrointestinal tract. These cells’ roles in functions like pacemaker activity, slow wave propagation, neural transmission, and mechanosensitivity, as well as their potential link to gastrointestinal motility disorders, were central to this research. Over the 11 years studies have intensively explored ICC’s structure, molecular and electrophysiological properties, and their significance in neurogastroenterology.

### 3.7 Burst detection of keywords and references

**Figure 9:**
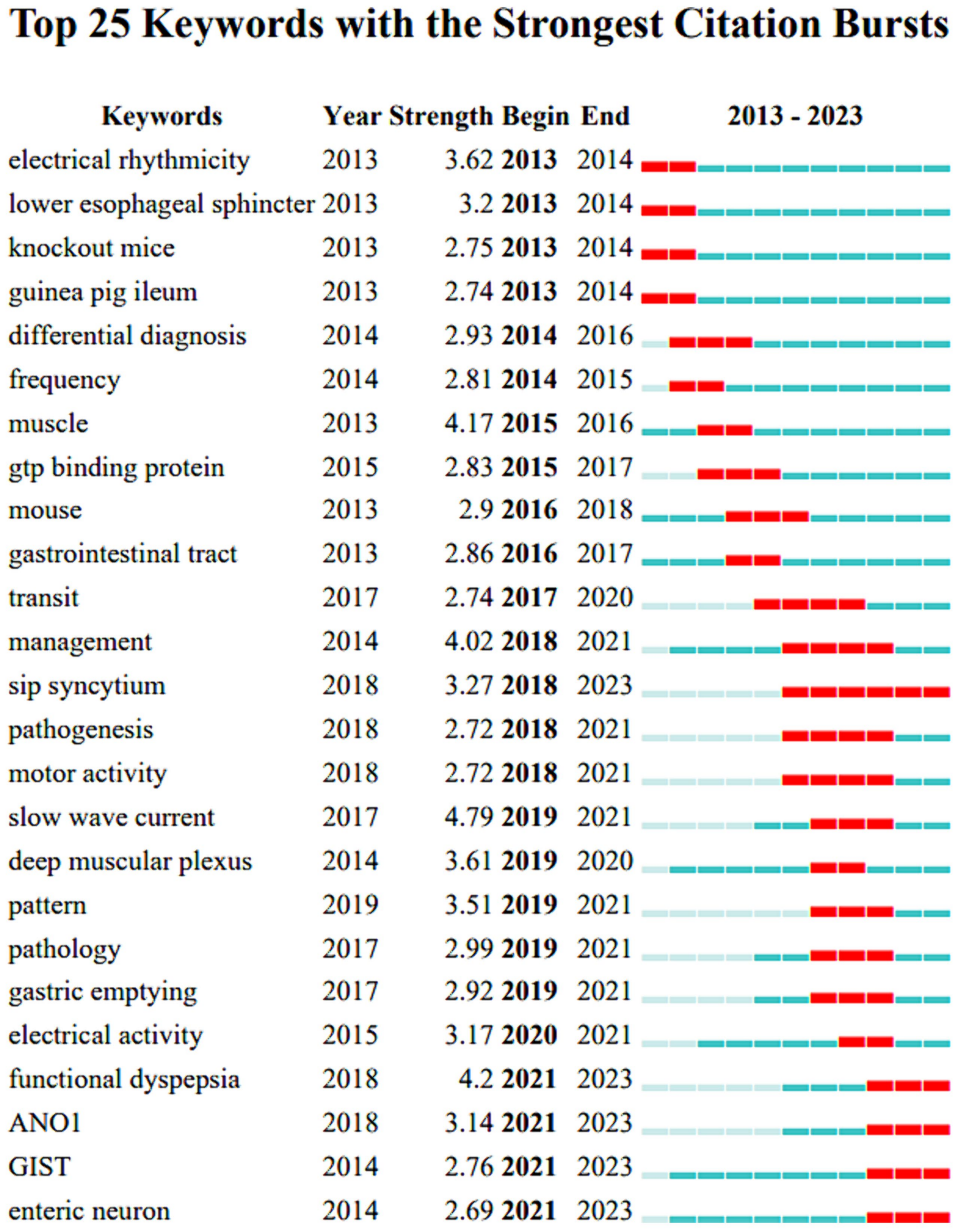
Burst detection of keywords.

Keywords serve as concise summaries of research content. By analyzing their development trends, we gained insights into the hotspots and focal points of specific research areas, which in turn informed research directions. The keyword burst analysis depicted in Figure 9 offered a broad overview of the evolution of ICC research hotspots over the past 11 years.

Specifically: 2013–2015: during this period researchers focused on ICC studies encompassing electrophysiology gene editing animal models and the diagnosis of related diseases

2016–2020: during this timeframe, there was a notable enhancement in the number of keywords, signifying an expanding range of research interests that covered diverse aspects, such as physiology, pathology, and regulation.

2021–2023: the primary emphasis during this period was on functional gastrointestinal disorders, the investigation of ANO1, and the interactions between ICC and enteric neurons.

**Figure 10:**
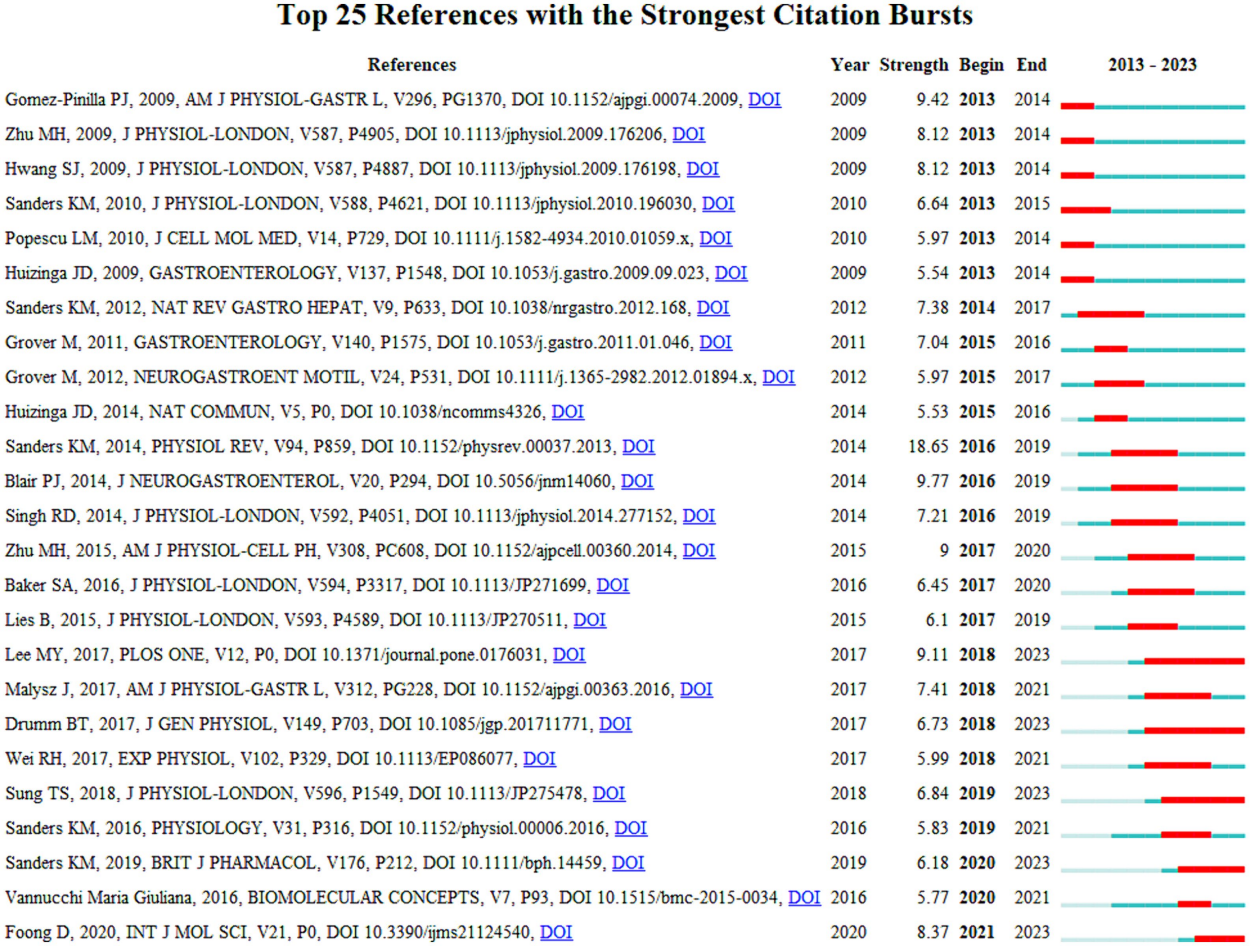
Burst detection of references.

As time progresses, the detection of citation bursts in reference literature provides insights into shifts in research focus. “Burst strength” refers to the citation burst intensity of a document. This value indicates the extent to which the number of citations for a particular document increases suddenly over a given period of time, compared to the average level (32). Among the top 25 cited references (Figure 10) in recent years, two highly cited articles stood out. One article, authored by Lee et al. (33), exhibited a burst strength of 9.11. Another article, written by Sung et al. (34), demonstrated a burst strength of 6.84. The groundbreaking study by Lee et al. utilized copGFP-labeled ICC mice and flow cytometry to successfully isolate populations of ICC from the mouse small intestine and colon, obtaining their transcriptome data. In addition, the authors constructed an interactive ICC genome browser based on the UCSC genome database, which provides a valuable reference for future functional studies. Sung et al. have made significant contributions to our understanding of the importance of enteric neural transmission in gastrointestinal motility and related mechanisms. The study shows that cholinergic nerve fibers are closely associated with interstitial cells of Cajal (ICC-IM) and mediate the electrical and mechanical responses to neural stimuli through the activation of the calcium-activated chloride channel anoctamin-1 (ANO1). Experimental results demonstrated that in wild-type mice, neural stimulation induced excitatory junction potentials and mechanical responses, whereas these responses were greatly reduced or eliminated in ANO1 knockout/downregulated mice. Furthermore, pharmacological blockade of ANO1 also inhibited these responses. The study further revealed that smooth muscle cells (SMC) express other receptors and ion channels associated with nerve stimulation. These findings highlight the deepening exploration of ICC by scholars, leading to the discovery of additional molecular mechanisms.

## 4 Discussion

### 4.1 General information

Our research utilized quantitative analysis tools, specifically CiteSpace and VOSviewer, to conduct an extensive investigation of the literature pertaining to ICC over the past 11 years. In addition, we conducted a comprehensive review of the research accomplishments and progress made in this area. Our study meticulously measured key aspects, including the annual publication count, geographical distribution, author collaboration networks, institutional affiliations, and journal evaluations. In the period from 2013 to 2023, there were minor fluctuations in the quantity of scholarly articles in the ICC field. However, overall, there was a consistently high level of publishing activity. Each year, about 80 papers were published, highlighting the sustained and widespread interest in the ICC field.

A geographical analysis of country/region and institution distribution revealed active engagement in ICC research from numerous countries and regions. Particularly, the China, United States, and South Korea displayed exceptional performance, accounting for a combined contribution of 60.83% of the total published papers. It was worth noting and emphasizing that publications from the United States achieved notable citation counts, highlighting strong collaborative relationships with other nations and emphasizing the extensive research conducted by the United States in the realm of ICC.

Among the top 10 ranked institutions, three were from the United States, three from China, and two from South Korea. Notably, the University of Nevada stood out as the most productive institution, having published the highest number of papers. Additionally, the Mayo Clinic received the highest ranking in total link strength, further highlighting the potential for significant impact resulting from its research outcomes. The strong collaborative relationships between countries and institutions contributed to breaking down academic barriers and promoting the development of ICC-related research.

The assessment and ranking of cited journals held great importance for researchers, as they aided in quickly identifying the most suitable journals for manuscript submissions. Moreover, analyzing citation frequencies facilitated the identification of primary research directions within the field. Most journals within the top 10 rankings boasted an IF exceeding 4.0, indicating a relatively high research quality in this domain. ·Noteworthy publications in esteemed journals such as the Journal of Physiology-London, Gastroenterology, Neurogastroenterology and Motility, and the American Journal of Physiology-Gastrointestinal and Liver Physiology demonstrated exceptional citation performance. Due to the potential impact of ICC research, these leading scholarly journals garnered significant interest from scholars. Therefore, closely monitoring these journals was crucial to remain abreast of the latest research advancements and discoveries.

Lastly, our analysis of author collaboration networks revealed that while several academic teams emerged within the ICC field, the level of collaboration among these teams was relatively low. We strongly encouraged different academic teams to actively enhance scholarly exchanges and engage in collaborative discussions, aiming to collectively delve deeper into the development of the ICC field. Such collaborative efforts had the potential to accelerate progress in this area. Among the top 10 authors, Kenton M. Sanders (52, 5.84%) has the highest number of publications, followed by Byung Joo Kim (41, 4.60%), and Gianrico Farrugia (29, 3.25%). This observation highlights the notable contributions these three authors have made to the ICC field.

### 4.2 Hotspots and Frontiers

Keyword analysis provides insights into the current trends within the research field. Keyword burst refers to the phenomenon where a particular keyword experiences a sudden increase in activity and appears frequently in academic literature within a specific academic field or topic. Utilizing co-occurrence analysis of keywords, we have identified the primary research directions and hot topics in ICC, shedding light on the changing development patterns within the thematic structure (35). The following are potential research frontiers.

Anoctamin 1 (ANO1) is an ion channel protein belonging to the TMEM16 family, which consists of 10 members in mammals (ANO1 to ANO10) (36, 37). The Ca^2+^-activated Cl^−^ channel ANO1 in ICC plays a crucial role in regulating pacemaker activity and responses to intestinal neurotransmitters (38, 39). It is primarily expressed in epithelial cells, smooth muscle cells, and sensory neurons (39–41), forming calcium-activated chloride channels in the cell membrane, and is considered a more specific ICC marker than c-Kit, as it does not label mast cells (42, 43). ANO1 has been shown to be significantly expressed in ICC and generate spontaneous transient inward currents in ICC (38, 44), generating slow waves in intact gastrointestinal smooth muscle (36, 44). Elevated intracellular Ca^2+^ leads to the generation and propagation of pacemaker potentials that are amplified by activation of ANO1 channels (10, 41). Abnormal expression or dysfunction of ANO1 is associated with the pathogenesis of various diseases, including diabetic gastroparesis, congenital megacolon, gastroesophageal reflux, and chronic constipation (45–48). It has been shown that pharmacological inhibition or gene silencing of ANO1 can block slow waves in intestinal smooth muscle, reducing intestinal motility in patients with diarrhea (49). However, validating ANO1 as a therapeutic target remains a challenging task. Despite significant progress in understanding the distribution, expression, structure, and pathophysiological function of ANO1, selective modulators are urgently needed to validate its therapeutic potential.

The enteric nervous system (ENS) is a complex network comprised of neurons and glial cells, commonly referred to as the “second brain” (50). The ENS possesses the capacity to regulate digestion, absorption, and defense, as well as exert influence over smooth muscle contractions, secretions, and gastrointestinal blood flow (12). It represents a substantial and intricate neural system within the intestines, capable of functioning autonomously from the central nervous system to coordinate gastrointestinal activities (51). ICC actively contributes to gastrointestinal motility and neural transmission (52). ICC effectively generates slow waves through their interaction with enteric neurons and smooth muscle fibers, playing a direct role in the regulation of gastrointestinal peristalsis and consequently in the overall functionality of the digestive system. Moreover, ICCs are significantly involved in enteric neural transmission and possess mechanoreceptor activity. They exhibit sensitivity to mechanical changes through the detection of intestinal stretch and contraction, enabling the adjustment of neural signals to adapt to various physiological and mechanical conditions, thus maintaining intestinal homeostasis.

The gastrointestinal organs manifest spontaneous, non-neuronal electrical rhythmicity and mechanical rhythmicity (39). ICC generates slow waves (53) and regulates them through specific electrical pathways, including ANO1, Ca^2+^-activated Cl^−^channels, and Ca-V3.2 channels (34, 38). Consequently, smooth muscle electrical activity is influenced (54), forming the foundation for key movement patterns such as peristalsis and segmentation.

Recent investigations have brought to light the interaction between ICC in the smooth muscle layer of the gastrointestinal tract and PDGFRα-positive cells (cells expressing platelet-derived growth factor receptor alpha) with SMC through gap junctions (55–57), resulting in the formation of SIP (Smooth Muscle-Interstitial Cells of Cajal) corpuscles (58). These entities collectively oversee pacemaker activity, slow wave propagation, conduction of input from motor neurons, and mechanical sensitivity (8), thereby coordinating the contraction function of SMC. Gap junctions induce changes in the excitability of ICC, consequently impacting the excitability of SMC. This facet assumes paramount importance in the regulation of gastrointestinal motility (59). ICC and PDGFRα cells express Ca^2+^-related conductance features: ICC generate inward currents via activated Cl^−^ channels encoded by ANO1, whereas PDGFRα cells express Ca^2+^-activated K^+^ channels encoded by Kcnn3, which generate outward currents that control calcium release and intracellular calcium transients (60). Neuronal transmission influences these transients, triggering activation of voltage-dependent calcium channels in the pacemaker ICC, maintaining depolarization during slow wave periods (38). These cells regulate peristalsis by activating calcium ion channels and ion channels, and are modulated by neuronal inputs (55). ICC also serve as pacemakers, generating slow waves that form the electrophysiologic basis of gastrointestinal motility. Any factor that causes morphological or functional changes in ICC or PDGFRα+ cells may affect the relative balance between ICC-ANO1-SMC and PDGFRα+ cells-SK3-SMC, resulting in abnormal gastrointestinal motility (61). Recent research proposes that the term “SIP genic” provides a more precise description of gastrointestinal dynamics regulation compared to the traditional term “myogenic” (60). The calcium handling mechanisms are central to mesenchymal cell function, yet the dynamics of these cells remain incompletely understood in gastrointestinal motility disorders. Nonetheless, the physiological and pathophysiological roles of these cells remain largely undefined in most cases. Further research is warranted to explore the forefront issues in this field.

Gastrointestinal stromal tumors (GIST) are rare tumors that originate in the interstitial cells of Cajal (62). Two-thirds of adult patients have c-Kit mutations, while a small percentage have PDGFRA mutations (63, 64). In recent years, the treatment of GIST has attracted much attention, mainly including targeted therapy and surgical resection. Imatinib has been widely used as first-line treatment for metastatic GIST, but patients usually experience disease progression after 2–3 years (65). Second- and third-line treatment options are limited, including sunitinib and regorafenib, but their efficacy is poor (66). Results from a large clinical trial indicated no significant difference in efficacy between avapritinib and regorafenib in patients with advanced GIST (67). A meta-analysis shows that neoadjuvant chemotherapy may improve 5 years overall survival, while local excision reduces hospitalization time (67). Recent studies have shown significant differences between GIST and wild-type (WT) KIT mutations, offering potential therapeutic perspectives and targets for overcoming imatinib resistance (69). Despite some progress in current therapeutic approaches, the outcome for certain GIST patients remains suboptimal. Therefore, it is necessary to further explore the potential role of ICC in the treatment of GIST, explore new therapeutic strategies and targets, to improve therapeutic efficacy and survival rates.

Functional dyspepsia (FD) is a chronic functional disorder originating from the gastroduodenal region, characterized by epigastric pain or burning sensation, postprandial fullness, or early satiety (70, 71). The Rome IV criteria classify FD into two distinct subtypes: postprandial distress syndrome (PDS) and epigastric pain syndrome (EPS) (72). Although the pathophysiology of FD remains uncertain, ICC are recognized as key regulatory mediators and therapeutic targets for the condition (73, 74). Several studies have suggested that both the quantity and dysfunction of ICC may contribute to the development of FD. Currently, treatment options for FD include dietary modifications, probiotics, antibiotics, acid suppressants, neuromodulators, prokinetics, and others, but none of these methods are consistently effective. Recent research has found that alterations in gut flora may play an important role in the pathogenesis of FD, especially changes in duodenal microbiome may be caused or contributed to by immune and neuronal dysregulation (71, 75, 76). Restoring microbial homeostasis with probiotics has been shown to be effective in FD (77, 78). Results of a meta-analysis suggest that spore-forming probiotics may be an effective treatment for FD patients, but more research is needed to validate their long-term efficacy and safety (79). However, the mechanisms by which gut microbiota influence gastrointestinal function and symptoms, and their association with ICC, remain unclear. Whether this process influences the amount and function of ICC may be a direction for future research.

### 4.3 Limitations

The application of bibliometrics relies on the availability of metadata, making the accuracy of the metadata a crucial factor (21). The analysis in this study was based on articles from the WoSCC database, while studies published in non-SCI journals or other databases were excluded, an omission that may have affected the accurate assessment of the study results. Also, we did not exclude the effect of journal self-citation rates, which may bias the results somewhat. It is important to note that CiteSpace and VOSviewer cannot completely replace systematic retrieval. Some bibliometric indicators, such as journal impact factors, may oversimplify the measurement of research outputs and fail to comprehensively reflect the research’s quality, innovation, and social impact. However, despite these limitations, they are expected to have a minor effect on the overall results and are unlikely to significantly alter the main trends proposed in this paper. In conclusion, this study serves as a foundation for understanding the research topics, hotspots, and development trends in ICC.

## 5 Conclusion

In this study, we conducted a bibliometric analysis to review the trends, hotspots, and frontiers of research related to ICC over the past 11 years. Our study identified 891 publications on ICC, revealing influential countries, institutions, and authors who have made significant contributions in this field. Additionally, we focused on specific topics to investigate research trends. According to our analysis, the role of ICC in the treatment of GIST and FD, as well as the relationship between ANO1, SIP syncytium, enteric neurons, and ICC, may become important directions for future research. Our analysis is particularly valuable to researchers in gastroenterology, oncology, and cell biology, providing insights that can guide future research directions.

## 6 Recommendations

This study presents the comprehensive exploration of ICC and its intricate interplay within the gastrointestinal system, providing valuable insights into current research trends. The identification of key areas and cutting-edge frontiers establishes a solid foundation for future investigations which focuses on NO, c-Kit receptor, ANO1, and the enteric nervous system. The elucidation of ICC’s role in gastrointestinal disorders underscores the significance of ICC, which is a potential therapeutic target. Further research on the physiological and pathophysiological aspects of ICC, especially in conditions like functional dyspepsia, holds great promise for advancing our understanding and developing targeted interventions.

## Author contributions

PL: Data curation, Formal analysis, Investigation, Methodology, Resources, Software, Visualization, Writing – original draft, Writing – review & editing. YaX: Data curation, Formal analysis, Resources, Validation, Visualization, Writing – original draft. LZ: Resources, Funding acquisition, Supervision, Writing – review & editing. XZ: Resources, Data curation, Formal analysis, Software, Validation, Writing – original draft. YiX: Funding acquisition, Project administration, Supervision, Writing – review & editing. XW: Funding acquisition, Project administration, Supervision, Writing – review & editing. MZ: Writing – review & editing, Data curation, Formal analysis, Software, Validation. XG: Funding acquisition, Project administration, Supervision, Writing – review & editing.
