# Corrigendum: A bibliometric analysis of interstitial cells of Cajal research

**DOI:** 10.3389/fmed.2024.1522574

**Published:** 2024-12-02

**Authors:** Pengyu Li, Yadan Xiao, Lan Zhou, Xuyuan Zhang, Yin Xu, Xiaojuan Wang, Menglong Zou, Xuan Guo

**Affiliations:** ^1^School of Integrated Chinese and Western Medicine, Hunan University of Chinese Medicine, Changsha, China; ^2^Integrated Traditional Chinese and Western Medicine Department, The Third Hospital of Changsha, Changsha, China; ^3^Department of Gastroenterology, The First Hospital of Hunan University of Chinese Medicine, Changsha, China; ^4^Science & Technology Innovation Center (National Key Laboratory Cultivation Base of Chinese Medicinal Powder & Innovative Medicinal Jointly Established by Province and Ministry), Hunan University of Chinese Medicine, Changsha, China

**Keywords:** interstitial cells of Cajal, CiteSpace, VOSviewer, bibliometrics, visualization

In the published article, there was an error in the article type. It was incorrectly published as a Review. The correct article type is: Systematic Review.

The authors apologize for this error and state that this does not change the scientific conclusions of the article in any way. The original article has been updated.

